# Epidemiological Analysis of Antinuclear Antibodies Positivity and Pattern Distribution in a Taiwanese Hospital-Based Cohort

**DOI:** 10.3389/bjbs.2025.15625

**Published:** 2025-12-15

**Authors:** Yu-Wei Tseng, Cheng-Li Lin, Si-Yu Chen, Tze-Kiong Er

**Affiliations:** 1 Division of Laboratory Medicine, Asia University Hospital, Asia University, Taichung, Taiwan; 2 Management Office for Health Data, China Medical University Hospital, Taichung, Taiwan; 3 Department of Nursing, Asia University, Taichung, Taiwan; 4 Department of Medical Laboratory Science and Biotechnology, Asia University, Taichung, Taiwan

**Keywords:** ANA patterns, antinuclear antibodies, autoimmune diseases, indirect immunofluorescence, prevalence

## Abstract

**Background:**

Autoimmune diseases pose an increasing global health burden. The detection of antinuclear antibodies (ANA) via indirect immunofluorescence (IIF) is central to diagnosing systemic autoimmune conditions. This study aimed to assess the prevalence and distribution of ANA patterns in a Taiwanese population.

**Methods:**

We conducted a retrospective, cross-sectional study of 8,299 patients who underwent ANA testing at Asia University Hospital between January 2021 and December 2023. ANA patterns were classified based on fluorescence staining characteristics. Demographic variables, including age and gender, were analyzed.

**Results:**

ANA positivity was observed in 35.3% of patients. The most frequent pattern was homogeneous (33.0%), followed by speckled (24.1%). Female patients had a significantly higher positivity rate (female-to-male ratio 2.7:1), and the ≥66-year age group accounted for 34.6% of ANA-positive cases. Mixed ANA patterns were identified in 22.8% of ANA-positive patients, with homogeneous-speckled being the most common combination (27.4%).

**Conclusion:**

This large-scale study provides valuable epidemiological data on ANA prevalence and pattern distribution in Taiwan. The predominance of homogeneous and speckled patterns, particularly among older female patients, aligns with established trends in autoimmune diseases. The high proportion of mixed ANA patterns suggests the need for further investigation into their clinical significance and diagnostic value.

## Introduction

Autoimmune diseases significantly impact individuals, families, and society, representing a growing public health challenge worldwide; they affect millions of people and lead to a variety of chronic health conditions, contributing to rising healthcare costs [1]. These disorders are characterized by the immune system mistakenly attacking the body’s own tissues, often leading to inflammation and organ damage [2]. The dramatic rise in autoimmunity and autoimmune diseases worldwide, likely driven by changes in environmental exposures, underscores the need for a deeper understanding of their diagnostic and therapeutic implications in both developed and developing countries [1, 3]. Autoimmune diseases are estimated to have an incidence rate ranging from 3.2% to 9.4%, with both incidence and prevalence showing a substantial increase over the past 30 years [4]. One of the key diagnostic markers in the early detection of autoimmune disorders is the presence of antinuclear antibodies (ANA) [5].

ANAs are a group of autoantibodies that target cellular nuclei, binding specifically to DNA, RNA, and protein-nucleic acid complexes; they serve as important markers for the diagnosis and activity of autoimmune diseases [6]. Systemic lupus erythematosus, Sjögren disease, and systemic sclerosis are heterogeneous chronic autoimmune conditions that primarily affect women, with antibodies commonly detected in patients with these systemic autoimmune diseases [7]. Females are at a higher risk for autoimmune diseases compared to males, with sex hormones playing a significant role in the underlying pathogenic mechanisms [8]. Autoantibodies are a hallmark feature in the classification of many autoimmune diseases, with ANA detection serving as a crucial step in diagnosing patients presenting with autoimmune symptoms, as it helps clinicians identify autoantibodies associated with a wide range of disorders [9].

Leong et al. conducted a nationwide population-based study of systemic lupus erythematosus (SLE) in Taiwan using the Longitudinal Health Insurance Database (2001–2011). They reported an overall prevalence of 8.11 per 10,000 in 2011, with markedly higher rates in women (14.3/10,000) compared to men (1.62/10,000). The highest prevalence occurred in women aged 40–49 years. The study also identified higher risks among men in rural areas and lower risks among individuals with higher income [10]. Recently, Mohamed-Ahmed et al. reported significant variation in the prevalence of autoimmune diseases among Chinese adults, which includes Mainland China, Hong Kong, Macau, and Taiwan. [11]. Their findings indicate that 2.7%–3.0% of the adult population, which is over 31 million individuals, is affected by one or more autoimmune conditions, a rate that is comparable to Western populations. The majority of these cases are attributed to autoimmune thyroid diseases and rheumatoid arthritis. Overall, both the incidence and prevalence of autoimmune diseases are rising significantly worldwide. This increase is likely due to multiple factors, including genetic predispositions, environmental influences, and enhanced awareness and diagnostic capabilities. Despite the escalating burden of autoimmune diseases in Taiwan, data on the frequency and patterns of ANA positivity in the local population remain limited.

The detection of ANA is typically carried out using indirect immunofluorescence (IIF) on HEp-2 cells, which allows for the visualization of various ANA patterns [12]. These patterns can provide insights into the underlying autoimmune condition and its severity. The diagnostic landscape for ANAs is shaped by the diverse patterns they exhibit under the microscope. Patterns such as homogeneous, speckled, centromeric, and nucleolar form the foundation of this diagnostic approach. A homogeneous pattern, often linked to antibodies targeting double-stranded DNA (dsDNA), is strongly associated with SLE. In contrast, speckled patterns may indicate the presence of antibodies against extractable nuclear antigens (ENAs) and are commonly linked to various autoimmune disorders. Patterns like nuclear speckled, nuclear homogeneous, nucleolar, and centromeric frequently appear in ANA-positive cases, with each pattern correlating to specific autoimmune conditions [13]. Alongside nuclear patterns, significant cytoplasmic and mitotic patterns can also be detected in HEp-2 IIFA analysis. Reporting these non-nuclear patterns is increasingly recognized for its clinical relevance [14].

Given the limited availability of detailed data on ANA patterns in Taiwan, this study aims to bridge this gap through a retrospective analysis of ANA-positive patients at our institution. The primary objectives are to evaluate the prevalence of ANA positivity and to characterize the distribution of ANA patterns within a hospital-based Taiwanese cohort. In addition, by analyzing demographic variables such as age and gender among ANA-positive individuals, we seek to identify factors potentially associated with autoimmune disease prevalence in this population. A better understanding of local ANA pattern distributions may enhance clinical interpretation, support more informed diagnostic decisions, and improve the management of patients with suspected autoimmune disorders.

## Materials and Methods

### Study Design and Population

This study was a retrospective, cross-sectional analysis conducted at the Asia University Hospital. The study included patients who were referred for ANA testing between January 2021 and December 2023. A total of 8,299 patients were enrolled, all of whom were suspected of having an autoimmune disorder based on clinical presentation. The age distribution of the patients ranged from 9 months (0.9 years) to 96.3 years, with a mean age of 51.8 years. Both male and female patients were included in the study, with no exclusion criteria based on age, sex, or clinical history. Inclusion criteria encompassed all patients referred for ANA testing during the study period, with their results reviewed; however, for patients with multiple test requests, only the initial ANA result was included, and subsequent results were excluded. This study was approved by the Institutional Review Board of China Medical University Hospital (IRB No. CMUH113-REC1-188) on November 22, 2024, and was conducted in accordance with the ethical standards of the institutional research committee and the 1964 Helsinki Declaration and its later amendments. Informed consent was waived by the IRB due to the retrospective and anonymized nature of the data.

### Sample Collection and Handling

Blood samples (4 mL) were collected from each patient using standard phlebotomy procedures. The samples were collected in clot activator vacuum tubes (yellow cap), and allowed to clot at room temperature for 30 min. The samples were then centrifuged at 1,300 × g for 10 min to separate the serum from the blood cells. The serum samples were immediately stored at 2 °C–8 °C and were processed within 48 h for ANA testing.

### ANA Detection by Indirect Immunofluorescence (IIF)

ANA testing was performed using the IIF method, which is widely regarded as the gold standard for ANA detection [15, 16]. We utilized the NOVA Lite® ANA HEp-2 kit (INOVA Diagnostics, USA), with HEp-2 cells serving as the substrate due to their high sensitivity and ability to produce distinct fluorescence patterns.

Patient serum samples were initially screened at a dilution of 1:80, a threshold commonly used in clinical laboratories and recommended by the kit manufacturer. Diluted samples were incubated with HEp-2 cells fixed on slides, followed by washing to remove unbound antibodies. A fluorescent-labeled secondary antibody was then added to detect bound ANA. Each slide was examined under a fluorescent microscope by two independent observers with over 5 years of experience in immunofluorescence analysis. Although we referred to the International Consensus on ANA Patterns (ICAP) framework for general guidance, ANA patterns in this study were categorized using conventional clinical pattern recognition prevalent in local practice, and without strict assignment to the standardized ICAP AC-nomenclature. The lack of adoption of the universal ICAP classification is a significant limitation of this study, as it impacts pattern standardization and global comparability.

In our laboratory, ANA results were reported as “negative” for titers <1:80 and “positive” for titers ≥1:80. Additional titration was performed for positive cases to identify endpoint titers at 1:160, 1:320, 1:640, 1:1280, and 1:2560. Staining patterns were classified into homogeneous, speckled, nucleolar, centromere, and cytoplasmic types. Mixed patterns were documented when more than one distinct staining pattern was simultaneously observed in a single sample.

### Statistical Analysis

Data were analyzed using SAS 9.4 (SAS Institute Inc., Cary, NC) and R software (R Foundation for Statistical Computing, Vienna, Austria). Descriptive statistics were employed to summarize patient demographics, ANA positivity rates, and the distribution of ANA patterns. A Chi-squared test of independence was conducted to compare the prevalence of ANA positivity across different age groups and genders, with a P-value <0.05 considered statistically significant.

## Results

### Demographic Characteristics of the Study Population

This retrospective analysis included a total of 8,299 patients who underwent ANA testing at our institution. Of these, 65.6% (n = 5,546) were female and 34.4% (n = 2,853) were male. The patients’ ages ranged from 9 months (0.9 years) to 96.3 years, with a mean age of 51.8 ± 17.5 years. The most represented age group was as follows, from youngest to oldest: ≤20 years (3.6%), 21–35 years (15.0%), 36–50 years (25.1%), 51–65 years (31.9%), and ≥66 years (24.5%).

**FIGURE 1:**
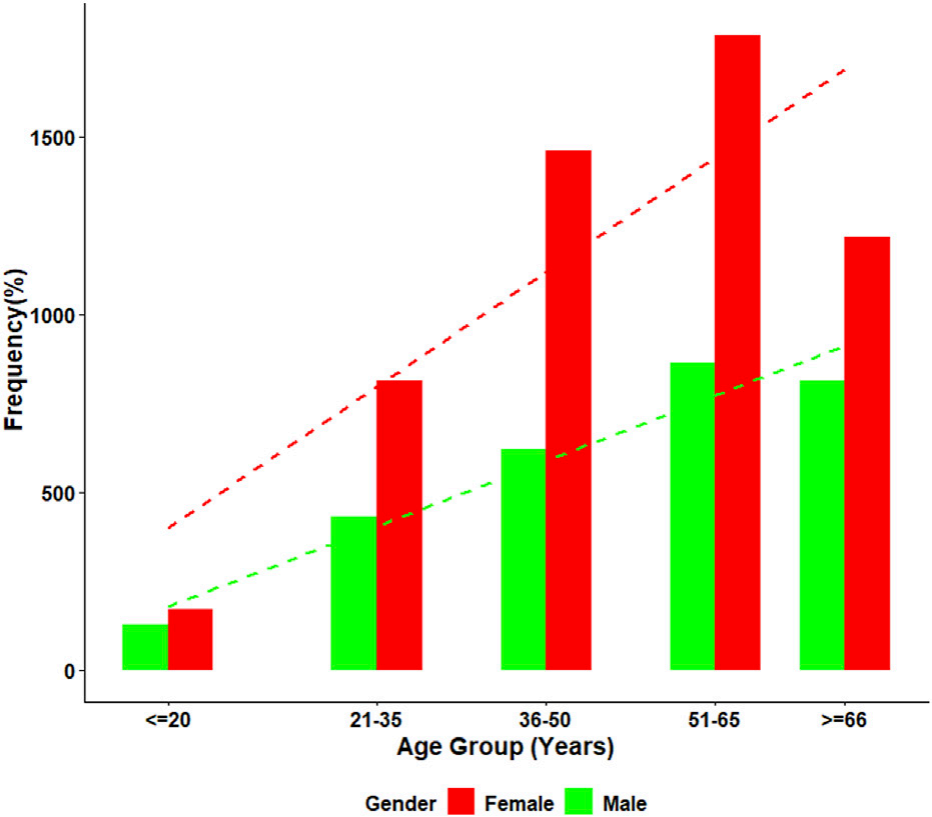
Age-specific distribution of ANA positivity by gender. This figure presents the percentage of ANA-positive individuals across five age groups for both males and females. Female participants showed consistently higher ANA positivity at all age intervals, with a clear increasing trend with age observed in both genders. Positivity rates rose markedly after age 50, reaching the highest levels in the ≥66-year group.

ANA positivity, defined as a titer of ≥1:80, was observed in 35.3% (n = 2,925) of the cohort. The detailed distribution of age, sex, and ANA positivity rates is presented in Figure 1. Statistical analysis using the Chi-squared test of independence demonstrated significant differences in ANA positivity across sex and age groups (all *p* < 0.001).

It is important to acknowledge that the study cohort represents a selected population referred for ANA testing due to clinical suspicion of autoimmune disease. Therefore, the reported prevalence figures should be interpreted in the context of this selection bias and may not reflect the general population.

### ANA Patterns

**FIGURE 2:**
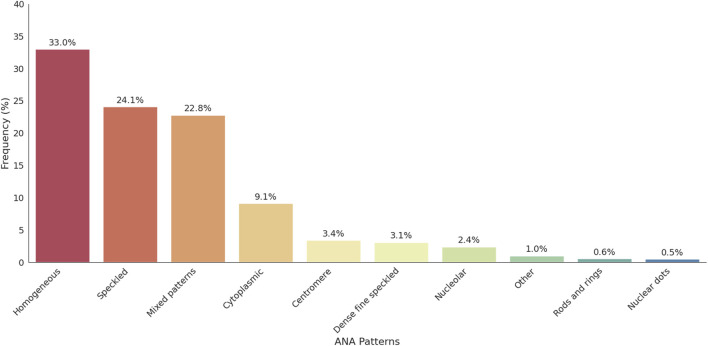
Distribution of ANA patterns among ANA-positive individuals. This figure illustrates the frequency of nuclear and cytoplasmic ANA patterns identified in the cohort. Homogeneous and speckled patterns were the most prevalent, followed by cytoplasmic and mixed patterns. ANA patterns with a frequency below 0.5% were grouped under the “Other” category for clarity; this includes centrioles, discrete dots, and other rare patterns.

**FIGURE 3:**
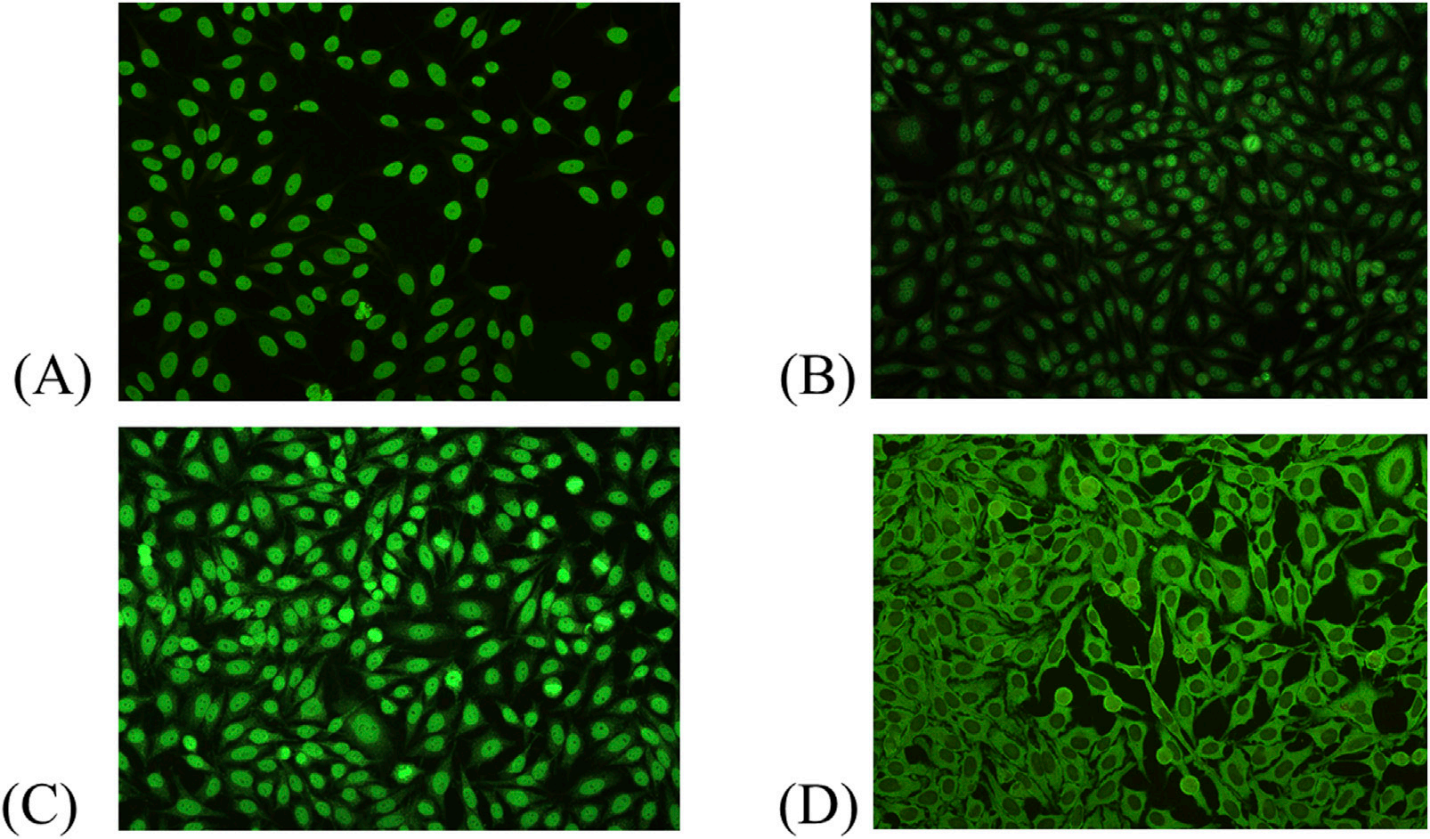
Representative ANA indirect immunofluorescence (IIF) patterns observed in this study. **(A)** Homogeneous pattern. **(B)** Speckled pattern. **(C)** Mixed pattern (homogeneous–cytoplasmic). **(D)** Cytoplasmic dense fine speckled pattern.

Among the 2,925 ANA-positive patients, the most frequently observed ANA pattern was the homogeneous pattern, accounting for 33.0% of cases. This was followed by the speckled pattern (24.1%), mixed patterns (22.8%), cytoplasmic pattern (9.1%), centromere pattern (3.4%), and dense fine speckled (DFS) pattern (3.1%). Pattern interpretation was primarily based on visual assessment during routine laboratory practice, though classification generally aligns with the ICAP. The distribution of ANA patterns is illustrated in Figure 2. Representative ANA IIF images corresponding to the major patterns observed in this study—including homogeneous, speckled, mixed homogeneous–cytoplasmic, and cytoplasmic dense fine speckled—are shown in Figure 3.

### Mixed ANA Patterns

**FIGURE 4:**
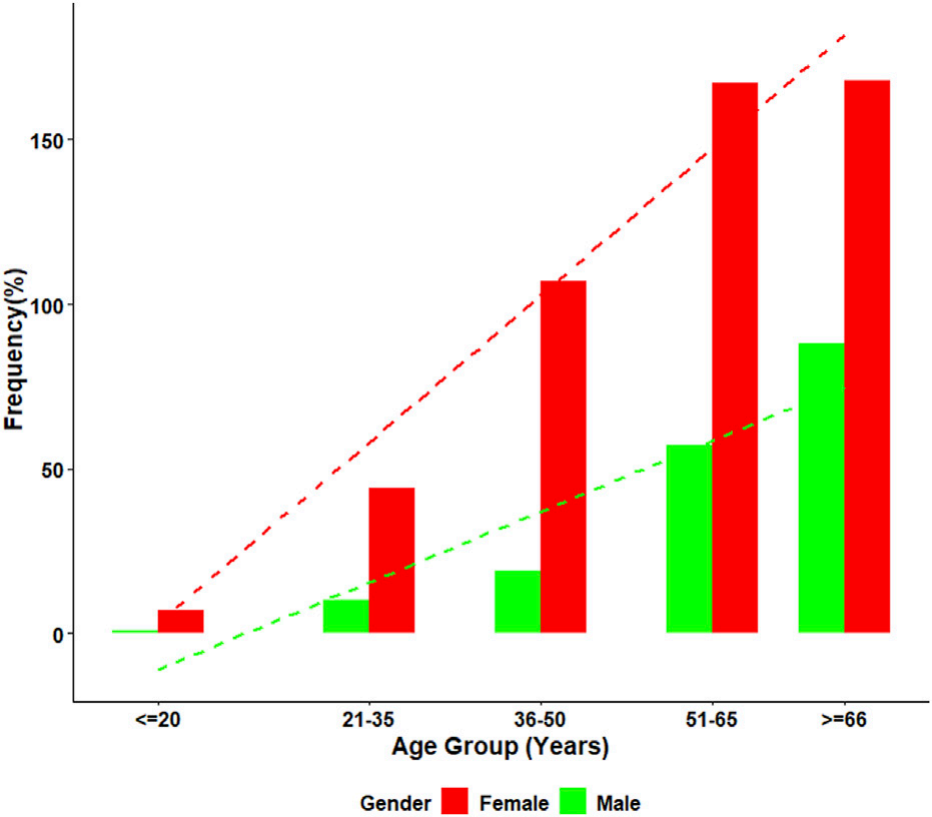
Distribution of mixed ANA patterns among ANA-positive individuals. This figure displays the distribution of mixed ANA patterns identified among ANA-positive patients. The most common combinations include homogeneous-speckled, homogeneous-cytoplasmic, and speckled-cytoplasmic patterns, emphasizing the diversity of mixed patterns observed in the study.

**FIGURE 5:**
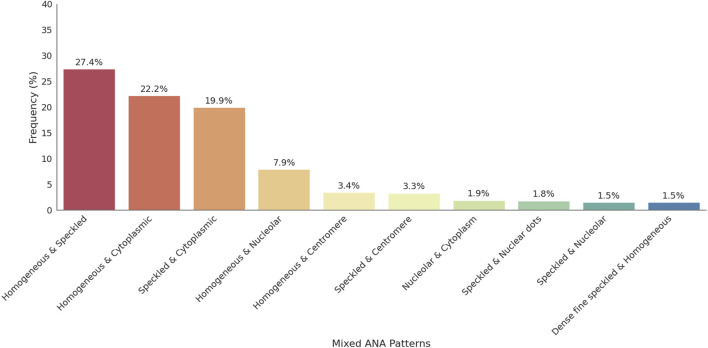
Age and gender distribution among individuals with mixed ANA patterns. This figure illustrates the percentage of individuals with mixed ANA patterns across five age groups, stratified by gender. Female patients consistently demonstrated higher frequencies of mixed ANA patterns compared with males at all ages. A clear age-related increase was observed in both sexes, with the highest proportion occurring in individuals aged ≥66 years.

Mixed ANA patterns were observed in 22.8% of the ANA-positive patients. The most frequently observed mixed ANA patterns were homogenous and speckled (183/688, 27.4%), homogenous and cytoplasmic (148/688, 22.2%), speckled and cytoplasmic (133/688, 19.9%), and homogenous and nucleolar (53/688, 7.9%). The distribution of mixed ANA patterns was significantly higher in females, with a female-to-male ratio of approximately 2.8:1 (493 females, 175 males). The highest prevalence of mixed ANA patterns was observed in the ≥66 years old age group. Figure 4 presents the frequency of mixed patterns observed among ANA-positive patients. Figure 5 summarizes the demographic characteristics of patients with mixed ANA patterns.

### ANA Titers

The titers of ANA-positive patients were distributed as follows: positive (1:80) in 47.8% of cases, moderately positive (1:160–1:320) in 35.8% of cases, and strongly positive (≥1:640) in 16.5% of cases. The strongest positive titers were more commonly associated with speckled and homogenous patterns.

## Discussion

The findings of this study offer valuable insights into the prevalence and patterns of antinuclear antibody (ANA) positivity in a Taiwanese hospital-based cohort, with an observed positivity rate of 35.3%. This figure is comparable to rates reported in other regional patient cohorts, such as a 33% ANA positivity rate reported by Gupta et al. in Central India [17]. Globally, the prevalence of ANA-positivity varies considerably depending on the population, age, diagnostic criteria, and geographical factors, with reported rates in various studies ranging from 12.3% to 55%. A comprehensive comparison of ANA prevalence rates and most common patterns across diverse international cohorts is summarized in Table 1.

**TABLE 1:** Summary of ANA prevalence and patterns in selected global studies.

Study (Reference)	Population/Setting	ANA positivity rate (%)	Most common ANA pattern(s)
Current study	Taiwanese (hospital-based cohort)	35.3	Homogeneous (33.0%), speckled (24.1%)
Gupta et al. [17]	Central India (hospital-based, patient samples)	33	Nuclear speckled (24.0%), nuclear homogenous (21.2%)
Imran et al. [18]	Pakistan (hospital-based, patient samples)	55	Nuclear homogeneous (27.7%), nuclear speckled (26.5%)
Sebastian et al. [19]	South India (hospital-based, patient samples)	N/A	Homogenous (45.5%), speckled (35.6%)
Minz et al. [20]	North India (hospital-based, patient samples)	12.3 (mean)	Not explicitly specified
Mengeloglu et al. [21]	Bolu, Turkey (hospital-based, patient samples)	15.8	Coarse speckled (31.2%), nucleolar (18.0%)
Krzemień et al. [22]	Polish adult population (LIPIDOGEN2015 sub-study)	15	AC-2 dense fine speckled (50.0%), AC-21 Reticular/AMA (15.4%)
Frodlund et al. [23]	Regional Swedish register (SLE patients, prospective follow-up programme KLURING)	N/A	Homogenous (54.0%), speckled (22.0%)
Satoh et al. [24]	U.S. Population, (NHANES)	13.8	Nuclear patterns (84.6%), cytoplasmic patterns (21.8%)
Dinse et al. [25]	U.S. Population (NHANES)	15.9 (in 2011–2012)	Not explicitly specified

Our study revealed that the most common individual ANA patterns were the homogeneous pattern (33.0%) and the speckled pattern (24.1%) (Figure 2). This aligns with established international trends, where studies from South India, Turkey, and Pakistan also demonstrate the consistent dominance of these patterns. This consensus reinforces the diagnostic utility of the indirect immunofluorescence (IIF) assay for initial screening, which some studies suggest may suffice for initial screening, potentially reducing the need for more costly immunological tests [22]. The observed prevalence rates highlight significant variability. For instance, the high rate of 55% reported in the Pakistani cohort contrasts sharply with lower mean rates observed in nationwide population studies, such as 13.8% in the U.S. population and 12.3% in North India (Table 1).

Approximately 25% of the population has detectable levels of ANAs, which have been linked to factors such as older age, female sex, ancestry, and environmental exposures; around 12%–20% are ANA positive, with approximately 2.5% exhibiting significantly elevated levels [24, 26–28]. Cytoplasmic and mitotic ANA patterns remain underrecognized and underreported, warranting more systematic evaluation [5]. Future studies should correlate these patterns with clinical features, liver function tests, and disease-specific antibodies to determine their diagnostic relevance. In particular, the cytoplasmic reticular/AMA pattern (AC-21) is strongly associated with autoimmune liver diseases and may serve as an important marker in suspected cases [29]. Recently, Gambino et al., highlighted the distinct distribution of ANA patterns across various liver diseases, revealing notable associations with conditions such as autoimmune hepatitis (AIH) and primary biliary cholangitis (PBC). Specifically, the AC1 homogeneous was more commonly observed in AIH patients, while the AC21 Cytoplasmic reticular/AMA showed a strong correlation with both PBC and cryptogenic chronic hepatitis [30]. Our findings revealed that the frequency of cytoplasmic ANA patterns was 9.1% in our study population, and further analysis in future studies is needed to clarify their potential link to autoimmune liver diseases. In summary, various studies have reported ANA positivity rates across different populations, ranging from 12.3% to 55%.

Al-Mughales et al. reported that speckled (52.1%) and homogeneous (35.2%) ANA patterns were predominant in SLE patients in Saudi Arabia, with peripheral, mixed, and speckled patterns strongly associated with pathogenic immune markers, suggesting their potential prognostic significance. Notably, mixed ANA patterns accounted for 6.5% of their cohort, reflecting a lower prevalence than single patterns but underscore their clinical relevance [31]. In a Polish cohort, AC-2 Dense Fine Speckled (50%) and AC-21 Reticular/AMA (14.4%) were the most common patterns, with ANA positivity more frequent in females (72%) and in patients over 50 years of age (64%) [22]. Similarly, a Swedish study involving 219 patients found homogeneous (54.3%) and speckled (22.4%) patterns to be most prevalent, while mixed homogeneous-speckled patterns represented 11.0% of cases [23]. In India, the nuclear pattern was most frequently observed (68.7%), especially the nuclear speckled (24%) and homogeneous (21.2%) subtypes. Cytoplasmic patterns were also present in 18.9% of patients, with cytoplasmic speckled comprising 8.4% [17]. Ramachandran et al. similarly reported a predominance of nuclear speckled (52.9%), followed by homogeneous (27.5%), mixed (13.7%), and cytoplasmic speckled (5.9%) patterns in their cohort [32]. Vermeersch et al. analyzed 9,268 ANA-positive samples and found homogeneous nuclear (36.5%), nuclear fine speckled (19.9%), and nucleolar (17%) patterns most frequently, with cytoplasmic and cell cycle–related patterns being less common [33]. In our study, mixed ANA patterns were observed in 22.8% of ANA-positive patients, with homogeneous-speckled combinations being the most frequent (183/688, 27.4%) (Figure 3). Collectively, these studies highlight speckled and homogeneous patterns as the most common across populations, while also emphasizing the importance of mixed patterns in autoimmune diagnostics. Further research incorporating antibody specificity and clinical correlation is essential to clarify their diagnostic and prognostic utility.

The female-to-male ratio of ANA positivity in our study was 2.7:1, which aligns with global data indicating that autoimmune diseases disproportionately affect women [34]. This pattern persisted across all age groups, with the highest rate of ANA positivity observed in the ≥66 age group, where 34.6% of patients tested positive. Hormonal and immunological differences between men and women have been widely studied [35], with some researchers suggesting that estrogen plays a role in modulating the immune response and increasing susceptibility to autoimmune diseases in females [36]. Furthermore, Meier et al. reported that ANA prevalence increases with age in the general population, and shorter telomere length, a marker of advanced cellular aging, are associated with the onset of ANA positivity [37]. A previous study estimated ANA prevalence at 15% in men and 22% in women over the age of 70 in the United States, nearly double the prevalence observed in individuals aged 12–19 [24]. Similarly, we found that the ≥66 age group exhibited the highest prevalence of ANA positivity.

One of the major strengths of this study is its large sample size, which provides a robust and comprehensive three-year overview of ANA patterns in a hospital-based Taiwanese population. However, several limitations must be acknowledged. First, because ANA testing is typically ordered for patients with clinical suspicion of autoimmune disease, the observed ANA positivity rate (35.3%) likely overestimates the true prevalence in the general population, and the findings should not be extrapolated beyond the hospital setting. Second, the retrospective single-center design limits the generalizability of our results to the broader Taiwanese population. Third, the lack of clinical data at the time of testing and absence of follow-up information restrict our ability to associate specific ANA patterns with definite disease phenotypes or outcomes. Additionally, because this retrospective dataset did not include clinical diagnoses or follow-up information, we were unable to correlate ANA positivity or specific ANA patterns with confirmed autoimmune diseases, which limits the interpretation of their diagnostic implications. Future multi-center prospective studies with clinical and serological correlation are needed.

Future prospective studies should incorporate diverse patient populations across multiple centers and include longitudinal clinical and immunological data to better elucidate the diagnostic and prognostic value of ANA patterns. Despite these limitations, our study contributes valuable epidemiological insights, particularly by highlighting the distribution of ANA patterns in specific demographic groups (e.g., older females). These findings may assist clinicians in prioritizing further autoimmune evaluations. For instance, the identification of mixed or cytoplasmic patterns could prompt additional testing for overlapping autoimmune syndromes or autoimmune liver disorders.

In conclusion, this study offers valuable epidemiological insights into the prevalence and patterns of ANA positivity in Taiwan, finding the homogeneous pattern to be the most common. ANA positivity was clearly more prevalent in female patients and older age groups, underscoring its diagnostic importance in autoimmune diseases. These findings highlight the need for future prospective, multicenter studies that incorporate longitudinal clinical and immunological data to better elucidate the diagnostic and prognostic value of ANA patterns. Such research is essential to explore the clinical significance of high-frequency mixed and cytoplasmic patterns and their potential to improve diagnostic accuracy and patient management.

## Data Availability

The raw data supporting the conclusions of this article will be made available by the authors, without undue reservation.
